# Hierarchical selection of genetic and gene by environment interaction effects in high-dimensional mixed models

**DOI:** 10.1177/09622802241293768

**Published:** 2024-12-10

**Authors:** Julien St-Pierre, Karim Oualkacha, Sahir Rai Bhatnagar

**Affiliations:** 1 Department of Epidemiology, Biostatistics and Occupational Health, 5620 McGill University, Montreal, QC, Canada; 2 Département de Mathématiques, Faculté des Sciences, Université du Québec à Montréal, Montreal, QC, Canada

**Keywords:** Gene by environment interaction, hierarchical variable selection, mixed effects model, high-dimensional data, statistical genetics

## Abstract

Interactions between genes and environmental factors may play a key role in the etiology of many common disorders. Several regularized generalized linear models have been proposed for hierarchical selection of gene by environment interaction effects, where a gene-environment interaction effect is selected only if the corresponding genetic main effect is also selected in the model. However, none of these methods allow to include random effects to account for population structure, subject relatedness and shared environmental exposure. In this article, we develop a unified approach based on regularized penalized quasi-likelihood estimation to perform hierarchical selection of gene-environment interaction effects in sparse regularized mixed models. We compare the selection and prediction accuracy of our proposed model with existing methods through simulations under the presence of population structure and shared environmental exposure. We show that for all simulation scenarios, including and additional random effect to account for the shared environmental exposure reduces the false positive rate and false discovery rate of our proposed method for selection of both gene-environment interaction and main effects. Using the $F_{1}$ score as a balanced measure of the false discovery rate and true positive rate, we further show that in the hierarchical simulation scenarios, our method outperforms other methods for retrieving important gene-environment interaction effects. Finally, we apply our method to a real data application using the Orofacial Pain: Prospective Evaluation and Risk Assessment (OPPERA) study, and found that our method retrieves previously reported significant loci.

## 1. Introduction

Genome-wide association studies (GWASs) have led to the identification of hundreds of common genetic variants, or single nucleotide polymorphisms (SNPs), associated with complex traits^1^ and are typically conducted by testing association on each SNP independently. However, these studies are plagued with the multiple testing burden that limits discovery of potentially important predictors, as genome-wide significance p -value threshold of $5\times 10^{-8}$ has become the standard. Moreover, GWASs have brought to light the problem of missing heritability, that is, identified variants only explain a low fraction of the total observed variability for traits under study.^2^ Beyond the identified genetic variants, interactions between genes and environmental factors may play a key role in the multifactorial etiology of many complex diseases that are subject to both genetic and environmental risk factors. For example, in assessing interactions between a polygenic risk score (PRS) and non-genetic risk factors for young-onset breast cancers (YOBC), Shi et al.^3^ showed a decreased association between the PRS and YOBC risk for women who had ever used hormonal birth control, suggesting that environmental exposure might result in risk stratification by interacting with genetic factors. Thus, there is a rising interest for discovering gene-environment interaction (GEI) effects as they are fundamental to better understand the effect of environmental factors in disease and to increase risk prediction accuracy.^4^

Several regularized generalized linear models (GLMs) have been proposed for selection of both genetic and GEI effects in genetic association studies,^5–7^ but currently no such method allows to include any random effect to account for genetic similarity between subjects. Indeed, one can control for population structure and/or closer relatedness by including in the model a polygenic random effect with variance-covariance structure proportional to a kinship or genetic similarity matrix (GSM).^8^ However, because kinship is a high-dimensional process, it cannot be fully captured by including only a few principal components (PCs) as fixed effects in the model.^9^ Hence, while both PC analysis (PCA) and mixed models (MMs) share the same underlying model, MMs are more robust in the sense that they do not require distinguishing between the different types of confounders.^10^ Moreover, MMs alleviate the need to evaluate the optimal number of PCs to retain in the model as fixed effects.

Except for normal responses, the joint estimation of variance components and fixed effects in regularized models is challenging both from a computational and analytical point of view, as the marginal likelihood for a generalized linear mixed model (GLMM) has no analytical form. To address these challenges, penalized quasi-likelihood (PQL) estimation is conceptually attractive as under this method, random effects can be treated as fixed effects, which allows to perform regularized estimation of both fixed and random effects as in the GLM framework. The computational efficiency of multivariable methods for high-dimensional MMs rely on performing a single spectral or Cholesky decomposition of the covariance matrix to rotate the phenotype and design matrix such that the transformed data become uncorrelated. For very large sample sizes, computing these decompositions can be very burdensome, with complexity of $O(n^{3})$ , where n is the sample size. Secondly, to obtain regularized estimates for the genetic predictors and GEI effects in linear mixed models (LMMs), we need to perform matrix multiplications with complexity of $O(n^{2})$ and $O(n^{2}p)$ to rotate the phenotype and genotype matrices respectively, with p the number of genetic predictors. Even for moderately small cohorts, the number of predictors in GWAS is often greater than 1 million, such that the genotype matrix itself will require around one terabyte of space to be loaded in memory in a normal double-precision format.^11^ In PQL regularized models, by minimizing the objective function with respect to the fixed effects vector only, we need not rotate the genotype matrix as we are conditioning on the random effects vector estimate.

Several authors have proposed to combine PQL estimation in presence of sparsity by inducing regularization to perform joint selection of fixed and/or random effects in multivariable GLMMs.^12–14^ However, these methods were not developed to specifically address selection of GEI effects. Although it is possible to perform naive selection of fixed and GEI effects by simply considering interaction terms as additional predictors, the aforementioned methods are not tailored to perform hierarchical selection, where interaction terms are only allowed to be selected if their corresponding main effects are active (i.e. non-zero) in the model.^15^ Hierarchical variable selection of GEI effects is appealing both for increasing statistical power^16^ and for enhancing model interpretability because interaction terms that have large main effects are more likely to be retained in the model.

Population structure and closer relatedness may also cause dependence between gene and environment, leading to selection of spurious GEI effects.^17^ In the context of GWAS, Sul et al.^18^ showed that under the polygenic model, ignoring this dependence may largely increase the false positive rate of GEI statistics. They proposed introducing an additional random effect that captures the similarity of individuals due to polygenic GEI effects to account for the fact that individuals who are genetically related and who share a common environmental exposure are more closely related. To our knowledge, the spurious selection of GEI effects in regularized models due to the dependence between gene and shared environmental exposure has not been explored yet. Thus, further work is needed to develop sparse regularized GLMMs for hierarchical selection of GEI effects in genetic association studies, while explicitly accounting for the complex correlation structure between individuals that arises from both genetic and environmental factors.

In this article, we develop a unified approach based on regularized PQL estimation to perform hierarchical selection of GEI effects in sparse regularized logistic mixed models. Similar to Sul et al.,^18^ we use a random effect that captures population structure and closer relatedness through a genetic kinship matrix, and shared environmental exposure through a GxE kinship matrix. We propose to use a composite absolute penalty (CAP) for hierarchical variable selection^19^ to seek a sparse subset of genetic and GEI effects that gives an adequate fit to the data. We derive a proximal Newton-type algorithm with block coordinate descent for PQL estimation with mixed lasso and group lasso penalties, relying on our previous work to address computational challenges associated with regularized PQL estimation in high-dimensional data.^12^ We compare the prediction and selection accuracy of our proposed model with existing methods through simulations under the presence of population structure and environmental exposure. Finally, we also apply our method to a real data application using the Orofacial Pain: Prospective Evaluation and Risk Assessment (OPPERA) study cohort^20^ to study the sex-specific association between temporomandibular disorder (TMD) and genetic predictors.

## 2. Methodology

### 2.1. Model

We have the following GLMM

(1) $$g(\mu_{i})=\eta_{i}=Z_{i}\theta +D_{i}\alpha +G_{i}\beta +(D_{i}G_{i})\gamma +b_{i}$$

for i=1,…,n , where $\mu_{i}=E(y_{i}|Z_{i},G_{i},D_{i},b_{i})$ , $Z_{i}$ is a 1×m row vector of covariates for subject i , $G_{i}$ is a 1×p row vector of genotypes for subject i taking values {0,1,2} as the number of copies of the minor allele, $(\theta^{⊺},\beta^{⊺}{)}^{⊺}$ is a (m+p)×1 column vector of fixed covariate and additive genotype effects including the intercept, $D_{i}$ is the exposure of individual i to a binary or continous environmental factor D with fixed effect α , and $\gamma =(\gamma_{1},\gamma_{2},\ldots ,\gamma_{p}{)}^{⊺}\in R^{p}$ is the vector of fixed GEI effects. Thus, we have a total of 2p+m+1 coefficients. We assume that $b=(b_{1},\ldots ,b_{n}{)}^{⊺}∼N(0,\tau_{g}K+\tau_{d}K^{D})$ is an n×1 column vector of random effects, with $\tau =(\tau_{g},\tau_{d}{)}^{⊺}$ the variance components that account for the relatedness between individuals. K is a known GSM or kinship matrix and $K^{D}$ is an additional kinship matrix that describes how individuals are related both genetically and environmentally, because a pair of individuals who are genetically related and share the same environment exposure have a non-zero kinship coefficient. The kinship matrix $K^{D}$ corrects for the spurious association of GEI effects due to population structure and subjects relatedness, in the same way that the kinship matrix K corrects for population structure and subjects relatedness on the main effects. Thus, the matrix $K^{D}$ can be interpreted as the covariance matrix between individuals that captures the residual variance explained by the sum of many small GEI effects across the genome. For a binary exposure, we define $K_{ij}^{D}=K_{ij}$ if $D_{i}=D_{j}$ , and $K_{ij}^{D}=0$ otherwise. For a continuous exposure, one possibility is to set $K_{ij}^{D}=K_{ij}(1-d(D_{i},D_{j}))$ , where d is a metric with range [0,1] . The phenotypes $y_{i}$ ’s are assumed to be conditionally independent and identically distributed given $\left(Z_{i},G_{i},D_{i},b\right)$ and follow any exponential family distribution with canonical link function g(⋅) , mean $E(y_{i}|Z_{i},G_{i},D_{i},b)=\mu_{i}$ and variance $\text{Var}(y_{i}|Z_{i},G_{i},D_{i},b)=\phi a_{i}^{-1}\nu (\mu_{i}),$ where ϕ is a dispersion parameter, $a_{i}$ are known weights, and ν(⋅) is the variance function.

### 2.2. Regularized PQL estimation

In order to estimate the model parameters and perform variable selection, we use an approximation method to obtain an analytical closed form for the marginal likelihood of model (1). We propose to fit (1) using a PQL method,^12,21^ from where the log integrated quasi-likelihood function is equal to

(2) $$\ell_{PQL}(\Theta ,\phi ,\tau ;\tilde{b})=-\frac{1}{2}\text{log}\left|\left(\tau_{g}K+\tau_{d}K^{D}\right)W+I_{n}\right|+\sum_{i=1}^{n}ql_{i}(\Theta ;\tilde{b})-\frac{1}{2}{\tilde{b}}^{⊺}{\left(\tau_{g}K+\tau_{d}K^{D}\right)}^{-1}\tilde{b}$$

where $\Theta =(\theta^{⊺},\alpha ,\beta^{⊺},\gamma^{⊺}{)}^{⊺},W=\phi^{-1}\Delta^{-1}=\phi^{-1}\text{diag}\left\{\frac{a_{i}}{\nu (\mu_{i})[g^{\prime }(\mu_{i}{)}^{2}]}\right\}$ is a diagonal matrix containing weights for each observation, $ql_{i}(\Theta ;b)=\int_{y_{i}}^{\mu_{i}}\frac{a_{i}(y_{i}-\mu )}{\phi \nu (\mu )}d\mu$ is the quasi-likelihood for the i th individual given the random effects b , and $\tilde{b}$ is the solution which maximizes $\sum_{i=1}^{n}ql_{i}(\Theta ;b)-\frac{1}{2}b^{⊺}(\tau_{g}K+\tau_{d}K^{D}{)}^{-1}b$ .

In typical genome-wide studies, the number of genetic predictors is much greater than the number of observations ( p>n ), and the fixed effects parameter vector Θ becomes underdetermined when modeling p SNPs jointly. Moreover, we would like to induce a hierarchical structure, that is, a GEI effect can be present only if both exposure and genetic main effects are also included in the model. Thus, we propose to add a sparse group lasso penalty^22^ to the negative quasi-likelihood function in (2) to seek a sparse subset of genetic and GEI effects that gives an adequate fit to the data. Indeed, the sparse group lasso is part of the family of CAPs that can induce hierarchical variable selection.^19^ We define the following objective function $Q_{\lambda }$ which we seek to minimize with respect to (Θ,ϕ,τ) :

(3) $$Q_{\lambda }(\Theta ,\phi ,\tau ;\tilde{b}):=-\ell_{PQL}(\Theta ,\phi ,\tau ;\tilde{b})+(1-\rho )\lambda \sum_{j}\|(\beta_{j},\gamma_{j}){\|}_{2}+\rho \lambda \sum_{j}|\gamma_{j}|$$

where λ>0 controls the strength of the overall regularization and ρ∈[0,1) controls the relative sparsity of the GEI effects for each SNP. In our modeling approach, we do not penalize the environmental exposure fixed effect α . Thus, a value of ρ=0 is equivalent to a group lasso penalty where we only include a predictor in the model if both its main effect $\beta_{j}$ and GEI effect $\gamma_{j}$ are non-zero. A value of 0<ρ<1 is equivalent to a sparse group lasso penalty where main effects can be selected without their corresponding GEI effects due to the different strengths of penalization, but a GEI effect is still only included in the model if the corresponding main effect is non-zero.

### 2.3. Estimation of variance components

Jointly estimating the variance components $\tau_{g},\tau_{d}$ and scale parameter ϕ with the regression effects vector Θ and random effects vector b is a computationally challenging non-convex optimization problem. Updates for $\tau_{g},\tau_{d}$ and ϕ based on a majorization-minimization (MM) algorithm^23^ would require inverting three different n×n matrices, with complexity $O(n^{3})$ , at each iteration. Thus, even for moderately small sample sizes, this is not practicable for genome-wide studies. Instead, we propose a two-step method where variance components and scale parameter are estimated only once under the null association of no genetic effect, that is assuming β=γ=0 , using the average information restricted maximum likelihood (AI-REML) algorithm.^12,24^

### 2.4. Spectral decomposition of the random effects covariance matrix

Given ${\hat{\tau }}_{g},{\hat{\tau }}_{d}$ , and $\hat{\phi }$ estimated under the null, spectral decomposition of the random effects covariance matrix yields

(4) $$\begin{array}{rl} {\left({\hat{\tau }}_{g}K+{\hat{\tau }}_{d}K^{D}\right)}^{-1} & ={\left({U\Lambda U}^{⊺}\right)}^{-1}\\ & =U\Lambda^{-1}U^{⊺} \end{array}$$

where U is an orthonormal matrix of eigenvectors and Λ is a diagonal matrix of eigenvalues $\Lambda_{1}\geq \Lambda_{2}\geq \cdots \geq \Lambda_{n}>0$ when both K and $K^{D}$ are positive definite. In practice, if K is rank-deficient, one can replace it by $K+\epsilon I_{n}$ for ϵ>0 small, to ensure that both K and $K^{D}$ are positive definite.

Using (4) and assuming that the weights in W vary slowly with the conditional mean,^25^ minimizing (3) is now equivalent to

(5) $$\begin{array}{rl} \hat{\Theta } & =\underset{\Theta }{\text{argmin }}-\sum_{i=1}^{n}ql_{i}(\Theta ;\tilde{\delta })+\frac{1}{2}{\tilde{\delta }}^{⊺}\Lambda^{-1}\tilde{\delta }+(1-\rho )\lambda \sum_{j}\|(\beta_{j},\gamma_{j}){\|}_{2}+\rho \lambda \sum_{j}|\gamma_{j}|\\ & =\underset{\Theta }{\text{argmin }}f(\Theta ;\tilde{\delta })+g(\Theta ) \end{array}$$

where $\tilde{\delta }=U^{⊺}\tilde{b}$ is the minimizer of $f(\Theta ;\delta ):=-\sum_{i=1}^{n}ql_{i}(\Theta ;\delta )+\frac{1}{2}\delta^{⊺}\Lambda^{-1}\delta$ . Thus, iteratively solving (5) also requires updating the solution $\tilde{\delta }$ at each step until convergence. Conditioning on the previous solution for Θ , $\tilde{\delta }$ is obtained by minimizing a generalized ridge weighted least-squares (WLS) problem with $\Lambda^{-1}$ as the regularization matrix. Then, conditioning on $\tilde{\delta }$ , $\hat{\Theta }$ is found by minimizing a WLS problem with a sparse group lasso penalty. We present in Appendix A our proposed proximal Newton-type algorithm that cycles through updates of $\tilde{\delta }$ and Θ .

## 3. Simulation study

We first evaluated the performance of our proposed method, called pglmm, against that of a standard logistic lasso, using the Julia package *GLMNet* which wraps the Fortran code from the original R package *glmnet* .^26^ Then, among logistic models that impose hierarchical interactions, we compared our method with the *glinternet* ^7^ and *gesso* ^6^ models which are both implemented in R packages. The *glinternet* method relies on overlapping group lasso, and even though it is optimized for selection of gene by gene interactions in high-dimensional data, it is applicable for selection of GEI effects. An advantage of the method is that it only requires tuning a single parameter value. On the other hand, *gesso* uses a CAP penalty with a group $L_{\infty }$ norm (iCAP) to induce a hierarchical structure, and the default implementation fits solutions paths across a two-dimensional grid of tuning parameter values. For all methods, selection of the tuning parameters is performed by cross-validation. The default implementation for *glmnet* , *glinternet* and *pglmm* is to find the smallest value of the tuning parameter λ such that no predictor are selected in the model, and then to solve the penalized minimization problem over a grid of decreasing values of λ . For these three methods, we used a grid of 50 values of λ on the log10 scale with $\lambda_{min}=0.01\lambda_{max}$ , where $\lambda_{max}$ is chosen such that no predictors are selected in the model. In addition, for *pglmm* , we solved the penalized minimization problem over a grid of 10 values of the tuning parameter ρ evenly spaced from 0 to 0.9, fitting a total of 500 models. The default implementation for *gesso* is to solve the minimization problem over a 20 × 20 two-dimensional grid of the tuning parameters values $\lambda_{1},\lambda_{2}$ , starting from the smallest value such that all coefficients are zero, and setting $\lambda_{min}=0.1\lambda_{max}$ . Finally, for *glmnet* , *gesso* , and *glinternet* , population structure and environmental exposure is accounted for by adding the top 10 PCs of the kinship matrix as additional covariates.

### 3.1. Simulation model

We performed a total of 100 replications for each of our simulation scenarios, drawing anew genotypes and simulated traits, using real genotype data from a high quality harmonized set of 4097 whole genomes from the Human Genome Diversity Project (HGDP) and the 1000 Genomes Project (1000 G).^27^ At each replication, we sampled 10,000 candidate SNPs from the chromosome 21 and randomly selected 100 ( 1% ) to be causal. Let S be the set of candidate causal SNPs, with |S|=100 , then the causal SNPs fixed effects $\beta_{j}$ were generated from a Gaussian distribution $N(0,h_{S}^{2}\sigma^{2}/|S|)$ , where $h_{S}^{2}$ is the fraction of variance on the logit scale that is due to total additive genetic fixed effects. Let $S^{\prime }$ be the set of candidate causal SNPs, not necessarily overlapping with S , that have a non-zero GEI effect, with $|S^{\prime }|=50$ , then the GEI effects $\gamma_{j}$ were generated from a Gaussian distribution $N(0,h_{S^{\prime }}^{2}\sigma^{2}/|S^{\prime }|)$ , where $h_{S^{\prime }}^{2}$ is the fraction of variance on the logit scale that is due to total additive GEI fixed effects. Further, we simulated a random effect from a Gaussian distribution $\epsilon ∼N(0,h_{g}^{2}\sigma^{2}K+h_{d}^{2}\sigma^{2}K^{D})$ , where $h_{g}^{2}$ and $h_{d}^{2}$ are the fractions of variance explained by the polygenic and polygenic by environment effects, respectively. The kinship matrices K and $K^{D}$ were calculated using a set of 50,000 randomly sampled SNPs excluding the set of candidate SNPs, and PCs were obtained from the singular value decomposition of K . We simulated a covariate for age using a Normal distribution and used the sex covariate provided with the data as a proxy for environmental exposure. Then, for i=1,…,4097 , binary phenotypes were generated using the following model:

(6) $$\text{logit}(\pi )=\text{logit}(\pi_{0k})-\text{log}(1.3)\times \mathrm{Sex}+\text{log}(1.05)\mathrm{Age}/10+\sum_{j\in S}\beta_{j}{\tilde{G}}_{j}+\sum_{j\in S^{\prime }}\gamma_{j}\cdot (\mathrm{Sex}\times {\tilde{G}}_{j})+\epsilon$$

where $\pi_{0k}$ , for k=1,…,7 , was simulated using a U(0.1,0.9) distribution to specify a different prevalence for each population in Table 1 under the null, and ${\tilde{G}}_{j}$ is the j th column of the standardized genotype matrix ${\tilde{g}}_{ij}=(g_{ij}-2p_{i})/\sqrt{2p_{i}(1-p_{i})}$ and $p_{j}$ is the minor allele frequency (MAF) for the j th predictor.

**Table 1.** Number of samples by population for the high quality harmonized set of 4097 whole genomes from the Human Genome Diversity Project (HGDP) and the 1000 Genomes Project (1000 G).

Population	1000 genomes	HGDP	Total
African	879 (28%)	110 (12%)	989 (24%)
Admixed American	487 (15%)	62 (7%)	549 (13%)
Central/South Asian	599 (19%)	184 (20%)	783 (19%)
East Asian	583 (18%)	234 (25%)	817 (20%)
European	618 (20%)	153 (16%)	771 (19%)
Middle Eastern	0	158 (17%)	158 (4%)
Oceanian	0	30 (3%)	30 (1%)
Total	3166	931	4097
Unrelated individuals	2520	880	3400

In all simulation scenarios, we set $h_{S}^{2}=0.2$ and $h_{S^{\prime }}^{2}=0.1$ such that each of the main effects ( |S|=100 ) or GEI effects ( $|S^{\prime }|=50$ ) explains 0.2% of the total variability on the logit scale. We compared the methods when $h_{g}^{2}=0.2$ and $h_{d}^{2}=0.1$ (i.e. low polygenic effects with $\sigma^{2}=9$ ), and when $h_{g}^{2}=0.4$ and $h_{d}^{2}=0.2$ (i.e. high polygenic effects with $\sigma^{2}=35$ ), respectively. In the first simulation scenario, we induced a hierarchical structure for the simulated data by imposing $\gamma_{j}\neq 0\to \beta_{j}\neq 0$ for j=1,…,p , such that the total number of causal SNPs is equal to 100, with half of them having non-zero GEI effects. In the second simulation scenario, we repeated the simulations from the first scenario, but without enforcing any hierarchical structure, such that the number of causal SNPs is equal to 150, with 100 of them having non-zero main effects, and 50 having non-zero GEI effects.

### 3.2. Metrics

To compare the performance of all methods in discovering important genetic predictors and estimating their main and interaction effects, we define in this section the performance metrics that will be used. First, we define the model size as simply the number of non-zero coefficients estimated by a model, that is, $\sum_{j=1}^{p}I({\hat{\beta }}_{j}\neq 0)$ for the main effects, and $\sum_{j=1}^{p}I({\hat{\gamma }}_{j}\neq 0)$ for the GEI effects. The false positive rate (FPR) is defined as the number of non-causal predictors that are falsely identified as causal (false positives), divided by the total number of non-causal predictors. The true positive rate (TPR), also known as sensitivity or recall, is defined as the number of true causal predictors that are correctly identified (true positives), divided by the total number of causal predictors. The false discovery rate (FDR) is defined as the number of false positives divided by the total number of selected predictors in the model. Thus, while FPR and TPR measure the ability of a model to distinguish between causal and non-causal predictors, the FDR actually measures the proportion of predictors that are not causal among those declared significant. Moreover, in genetic association studies where the number of non-causal predictors is very high, we are more interested in controlling the FDR rather than the FPR. Alternatively, we can define the precision as 1 minus the FDR, which measures the proportion of causal predictors among those declared significant. The $F_{1}$ score is defined as the harmonic mean of the precision and TPR, and it can be used to take into account that methods with a large number of selected predictors will likely have a higher TPR, and inversely that methods with a lower number of selected predictors will likely have a higher precision. Finally, the area under the curve (AUC) is used as a measure of the predictive performance of all methods when predicting the binary status of individuals. It takes into account the TPR of all methods at various FPR values when making individual predictions. A higher AUC means that a method has a better capacity at distinguishing between cases and controls.

### 3.3. Results

**Figure 1.**
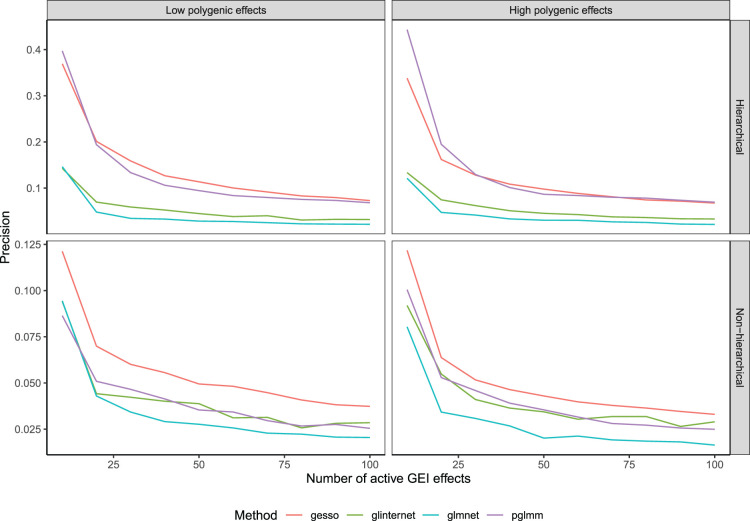
Precision of compared methods averaged over 100 replications as a function of the number of active gene-environment interaction (GEI) effects.

**Figure 2.**
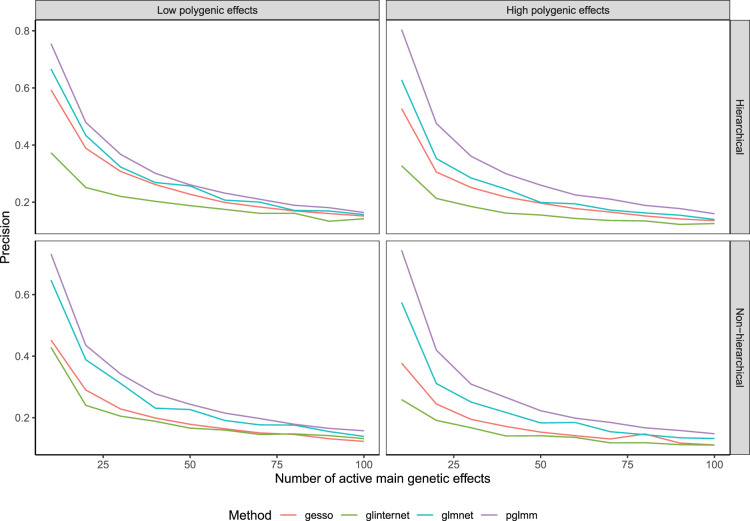
Precision of compared methods averaged over 100 replications as a function of the number of active main effects in the model.

We obtained solutions paths across a one dimensional (*glmnet*, *glinternet*) or two-dimensional grid of tuning parameter values ( *gesso* , *pglmm* ) for the hierarchical and non-hierarchical simulation scenarios and reported the mean precision, that is, the proportion of selected predictors that are causal, over 100 replications for the selection of GEI effects (Figure 1) and main genetic effects (Figure 2) respectively. We see from Figure 1 that in the hierarchical simulation scenario, *gesso* and *pglmm* retrieve important GEI effects with better precision than *glmnet* and *glinternet* . When we simulate a low random polygenic GEI effect, *gesso* slightly outperforms *pglmm* , but when we increase the heritability of the two random effects, both methods perform similarly. When we simulate data under no hierarchical assumption, precision for all hierarchical models fall drastically, although they still perform better than the standard lasso model. We note that *gesso* retrieves important GEI effects with equal or better precision than other methods in all simulation settings. This is explained by the fact that *gesso* is using a CAP penalty with $L_{\infty }$ group norm which has been shown to perform better than the sparse group lasso for retrieving interaction effects.^19^ On the other hand, we see from Figure 2 that *pglmm* outperforms all methods for retrieving important main effects for both hierarchical and non-hierarchical simulation scenarios. When we simulate low polygenic effects, *pglmm* and *glmnet* perform comparably. We also note that *gesso* retrieves main effects with less precision than *glmnet* and *pglmm* in all scenarios. At last, the precision of *glinternet* is considerably lower than all other methods until the number of selected main genetic effects in the model is large.

In practice, we often do not have any a priori knowledge for the number of main effects and/or GEI effects that we want to include in the final model. Thus, instead of comparing methods at a fixed number of selected predictors along their regularization paths, we used cross-validation to compare how each method performs when having to select an optimal number of predictors in the model for the same two simulation scenarios that we previously described. We randomly split the data ( n=4097 ) into training and test subjects, using a 80/20 ratio, and fitted the full lasso solution path on the training set for 100 replications. We report the model size, FPR, TPR, FDR, and $F_{1}$ score on the training sets, and the area under the ROC curve (AUC) when making predictions on the independent test subjects. To assess the potential spurious association of both main and GEI effects due to shared environmental exposure, we compare our method when including only the kinship matrix K (*plmm* (1 Random effect (RE))) and when including both K and $K^{D}$ matrices (pglmm (2 REs)).

With respect to selection of the GEI effects (Table 2), the comparative performance of each method varies depending on the simulation scenario. As expected, we see that including an additional random effect reduces the FPR for all simulation scenarios for our proposed method. Unsurprisingly, *glinternet* and *glmnet* have the lowest FPRs of all methods since they always select the least number of GEI effects in the final models. Consequently, they have the smallest TPRs in all scenarios. By using the $F_{1}$ score to account for the trade-off between FDR and TPR, we have that *pglmm* performs the best in hierarchical simulation scenarios, while *gesso* performs better in the non-hierarchical scenarios.

**Table 2.** Results for the GEI effects γ .

		Non-hierarchical model		Hierarchical model	
Metric	Method	Low ϵ	High ϵ	Low ϵ	High ϵ
Model size	*pglmm* (1 RE)	84.6	99.2	95.5	103
	*pglmm* (2 REs)	58.4	57.8	63.9	59.8
	*glmnet*	21.6	38.8	22.7	38.6
	*glinternet*	17.5	39.2	19.5	39.6
	*gesso*	64.3	102	78.6	110
FPR	*pglmm* (1 RE)	$8.31\times 10^{-3}$	$9.76\times 10^{-3}$	$8.96\times 10^{-3}$	$9.69\times 10^{-3}$
	*pglmm* (2 REs)	$5.72\times 10^{-3}$	$5.65\times 10^{-3}$	$6.00\times 10^{-3}$	$5.55\times 10^{-3}$
	*glmnet*	$2.09\times 10^{-3}$	$3.80\times 10^{-3}$	$2.20\times 10^{-3}$	$3.76\times 10^{-3}$
	*glinternet*	$1.68\times 10^{-3}$	$3.79\times 10^{-3}$	$1.84\times 10^{-3}$	$3.79\times 10^{-3}$
	*gesso*	$6.20\times 10^{-3}$	$9.94\times 10^{-3}$	$7.38\times 10^{-3}$	$1.06\times 10^{-2}$
TPR	*pglmm* (1 RE)	0.039	0.043	**0.126**	**0.124**
	*pglmm* (2 REs)	0.030	0.031	0.084	0.091
	*glmnet*	0.016	0.020	0.018	0.025
	*glinternet*	0.016	0.030	0.024	0.038
	*gesso*	**0.052**	**0.068**	0.104	0.103
FDR	*pglmm* (1 RE)	0.966	0.965	0.926	**0.908**
	*pglmm* (2 REs)	**0.936**	0.968	0.921	**0.889**
	*glmnet*	0.962	0.974	0.955	0.967
	*glinternet*	0.948	0.956	0.923	0.949
	*gesso*	0.945	**0.948**	**0.912**	0.930
$F_{1}$	*pglmm* (1 RE)	0.035	0.033	**0.091**	**0.084**
	*pglmm* (2 REs)	0.039	0.036	0.082	**0.090**
	*glmnet*	0.036	0.033	0.035	0.036
	*glinternet*	0.040	0.038	0.045	0.048
	*gesso*	**0.052**	**0.048**	0.083	0.067

*Note:* For each simulation scenario, we report the mean value over 100 replications when we simulate only one random effect with low heritability (low ϵ ) and we simulate two random effects with high heritability (high ϵ ). Bolded values indicate the method with the best performance according to each metric.

FPR: false positive rate; TPR: true positive rate; FDR: false discovery rate; RE: random effect.

Model size is defined as $\sum_{j=1}^{p}I({\hat{\gamma }}_{j}\neq 0)$ .

FPR is defined as $\sum_{j=1}^{p}I({\hat{\gamma }}_{j}\neq 0\;\cap \gamma_{j}=0)/\sum_{j=1}^{p}I(\gamma_{j}=0)$ .

TPR is defined as $\sum_{j=1}^{p}I({\hat{\gamma }}_{j}\neq 0\;\cap \gamma_{j}\neq 0)/\sum_{j=1}^{p}I(\gamma_{j}\neq 0)$ .

FDR is defined as $\sum_{j=1}^{p}I({\hat{\gamma }}_{j}\neq 0\;\cap \gamma_{j}=0)/\sum_{j=1}^{p}I({\hat{\gamma }}_{j}\neq 0)$ .

$F_{1}$ is defined as $2\times {\left(\frac{1}{1-FDR}+\frac{1}{TPR}\right)}^{-1}$ .

With respect to the genetic main effects (Table 3), *pglmm* selects the lowest number of predictors in the model, and thus has the lowest FPR and FDR in all simulation scenarios. Again, adding an additional random effect reduces the FPR for *pglmm* , but to a lower extent than for selection of GEI effects. On the other hand, *glinternet* always selects the largest number of predictors in all scenarios, and hence has the highest TPR and FPR values. Using the $F_{1}$ score to balance FDR and TPR, we see that *pglmm* performs the best for retrieving the important main genetic effects in all simulation scenarios. Also, we see that *gesso* and *pglmm* perform similarly when the heritability of the polygenic random effects is low, but when we increase the heritability, the FDR for *gesso* increases drastically, and the number of selected main effects becomes on average more than 1.5 times higher than for *pglmm* . Results for the accuracy of predicting binary outcomes in independent test sets are included in Table 4. We see that *pglmm* with two random effects outperforms all other methods for all simulation scenarios.

**Table 3.** Results for the genetic predictors main effects β .

		Non-hierarchical model		Hierarchical model	
Metric	Method	Low ϵ	High ϵ	Low ϵ	High ϵ
Model size	*pglmm* (1 RE)	227	212	220	204
	*pglmm* (2 REs)	206	190	214	179
	*glmnet*	278	444	286	450
	*glinternet*	299	481	312	480
	*gesso*	212	361	224	367
FPR	*pglmm* (1 RE)	$2.09\times 10^{-2}$	$1.96\times 10^{-2}$	$2.01\times 10^{-2}$	$1.87\times 10^{-2}$
	*pglmm* (2 REs)	$1.89\times 10^{-2}$	$1.74\times 10^{-2}$	$1.95\times 10^{-2}$	$1.62\times 10^{-2}$
	*glmnet*	$2.59\times 10^{-2}$	$4.24\times 10^{-2}$	$2.66\times 10^{-2}$	$4.29\times 10^{-2}$
	*glinternet*	$2.80\times 10^{-2}$	$4.61\times 10^{-2}$	$2.91\times 10^{-2}$	$4.57\times 10^{-2}$
	*gesso*	$1.95\times 10^{-2}$	$3.42\times 10^{-2}$	$2.05\times 10^{-2}$	$3.47\times 10^{-2}$
TPR	*pglmm* (1 RE)	0.195	0.181	0.208	0.196
	*pglmm* (2 REs)	0.188	0.177	0.206	0.188
	*glmnet*	0.215	0.244	0.226	0.257
	*glinternet*	**0.216**	**0.246**	**0.237**	**0.271**
	*gesso*	0.190	0.220	0.210	0.238
FDR	*pglmm* (1 RE)	**0.895**	**0.895**	**0.891**	**0.883**
	*pglmm* (2 REs)	**0.888**	**0.885**	**0.885**	**0.870**
	*glmnet*	0.920	0.944	0.917	0.942
	*glinternet*	0.925	0.948	0.922	0.943
	*gesso*	0.906	0.937	0.903	0.932
$F_{1}$	*pglmm* (1 RE)	**0.123**	**0.120**	**0.136**	**0.134**
	*pglmm* (2 REs)	**0.126**	**0.124**	**0.138**	**0.140**
	*glmnet*	0.115	0.091	0.119	0.094
	*glinternet*	0.109	0.085	0.116	0.094
	*gesso*	0.123	0.096	0.131	0.103

*Note:* For each simulation scenario, we report the mean value over 100 replications when we simulate two random effects with low heritability (low ϵ ) and high heritability (high ϵ ). Bolded values indicate the method with the best performance according to each metric.

FPR: false positive rate; TPR: true positive rate; FDR: false discovery rate; RE: random effect.

Model size is defined as $\sum_{j=1}^{p}I({\hat{\beta }}_{j}\neq 0)$ .

FPR is defined as $\sum_{j=1}^{p}I({\hat{\beta }}_{j}\neq 0\;\cap \beta_{j}=0)/\sum_{j=1}^{p}I(\beta_{j}=0)$ .

TPR is defined as $\sum_{j=1}^{p}I({\hat{\beta }}_{j}\neq 0\;\cap \beta_{j}\neq 0)/\sum_{j=1}^{p}I(\beta_{j}\neq 0)$ .

FDR is defined as $\sum_{j=1}^{p}I({\hat{\beta }}_{j}\neq 0\;\cap \beta_{j}=0)/\sum_{j=1}^{p}I({\hat{\beta }}_{j}\neq 0)$ .

$F_{1}$ is defined as $2\times {\left(\frac{1}{1-FDR}+\frac{1}{TPR}\right)}^{-1}$ .

**Table 4.** Results for the prediction accuracy of a binary outcome on test sets.

		Non-hierarchical model		Hierarchical model	
Metric	Method	Low ϵ	High ϵ	Low ϵ	High ϵ
AUC	*pglmm* (1 RE)	**0.719**	**0.786**	**0.728**	**0.788**
	*pglmm* (2 REs)	**0.723**	**0.790**	**0.730**	**0.792**
	*glmnet*	0.688	0.753	0.695	0.751
	*glinternet*	0.702	0.760	0.710	0.761
	*gesso*	0.695	0.750	0.707	0.751

*Note:* For each simulation scenario, we report the mean AUC value over 100 replications when we simulate two random effects with low heritability (low ϵ ) and high heritability (high ϵ ). Bolded values indicate the method with the best performance according to each metric.

RE: random effect; AUC: area under the curve.

## 4. Discovering sex-specific genetic predictors of painful temporomandibular disorder

Significant associations between temporomandibular disorder (TMD), which is a painful disease of the jaw, and four distinct loci have been previously reported in combined or sex-segregated analyses on the OPPERA study cohort.^28^ Moreover, TMD has much greater prevalence in females than in males and is believed to have some sex-specific pathophysiologic mechanisms.^29^ In this analysis, we wanted to explore the comparative performance of our proposed method *pglmm* in selecting important sex-specific predictors of TMD and its performance predicting the risk of painful TMD in independent subjects from two replication cohorts, the OPPERA II Chronic TMD Replication case-control study, and the Complex Persistent Pain Conditions (CPPC): Unique and Shared Pathways of Vulnerability study, using the OPPERA cohort as discovery cohort. Sample sizes and distribution of sex, cases and ancestry for the three studies are shown in Table 5, and further details on study design, recruitment, subject characteristics, and phenotyping for each study are provided in the Supplemental Material of Smith et al.^28^ (available at http://links.lww.com/PAIN/A688).

**Table 5.** Demographic data for the OPPERA training cohort, and for the OPPERA2 and CPPC test cohorts.

	Study name		
	OPPERA	OPPERA2	CPPC
*N* (% female)	3030 (64.6)	1342 (66.0)	390 (84.4)
Cases (%)	999 (33.0)	444 (33.0)	164 (42.0)
Ancestry (% white)	61	79	68

OPPERA: Orofacial Pain: Prospective Evaluation and Risk Assessment; CPPC: Complex Persistent Pain Conditions.

We used the imputed data described by Smith et al.^28^ Genotypes were imputed to the 1000 Genomes Project phase 3 reference panel using the software packages SHAPEIT^30^ for prephasing and IMPUTE version 2.^31^ For each cohort independently, we assessed imputation quality taking into account the number of minor alleles as well as the information score such that a SNP with rare MAF must pass a higher quality information threshold for inclusion. After merging all three cohorts, we tested for significant deviations of the Hardy-Weinberg equilibrium (HWE) separately in cases and controls, using a more strict *p*-value threshold for hypothesis testing among cases to avoid discarding disease-associated SNPs that are possibly under selection^32^ ( $<10^{-6}$ in controls, $<10^{-11}$ in cases). We filtered using a SNP call rate > 95% on the combined dataset to retain imputed variants present in all cohorts, which resulted in a total of 4.8 million imputed SNPs. PCs and kinship matrices were calculated on the merged genotype data using the --pca and --make-rel flags in PLINK,^33^ after using the same HWE *p*-value threshold and SNP call rate as for the imputed data. To reduce the number of candidate predictors in the regularized models, we performed a first screening by testing genome-wide association with TMD for subjects in the OPPERA discovery cohort using PLINK. We fitted a logistic regression for additive SNP effects, with age, sex, and enrollment site as covariates and the first 10 PCs to account for population stratification, and retained all SNPs with a *p*-value below 0.05, which resulted in a total of 243 thousand predictors.

We present in Table 6 the estimated odds ratios (OR) by each method, *pglmm* , *gesso* , and *glmnet* , for the selected SNPs for both main and GEI effects. Of note, it was not possible to use the *glinternet* package due to computational considerations, its memory requirement being too large for the joint analysis of the 243 thousand preselected predictors. All three methods selected the imputed insertion/deletion (indel) polymorphism on chromosome 4 at position 146,211,844 (rs5862730), which was the only reported SNP that reached genome-wide significance in the full OPPERA cohort ( OR=1.4 , 95% confidence interval (CI): [1.26;1.61] , $P=2.82\times 10^{-8}$ ).^28^ In a females-only analysis, rs5862730 was likewise associated with TMD ( OR=1.54 , 95% CI: [1.33;1.79] , $P=1.7\times 10^{-8}$ ), and both pglmm and gesso selected the GEI term between rs5862730 and sex.

**Table 6.** Selected SNPs by each method with their estimated odds ratios (OR) for the main effects ( β ) and GEI effects ( γ ) from the TMD real data analysis.

		*pglmm*			*gesso*			*glmnet*
Chromosome	Position	OR ${}_{\beta }$	OR ${}_{\gamma }$	OR ${}_{\beta +\gamma }$	OR ${}_{\beta }$	OR ${}_{\gamma }$	OR ${}_{\beta +\gamma }$	OR ${}_{\beta }$
3	5,046,726	–	–	–	1.0042	1.0087	1.0129	–
3	153,536,154	1.0020	–	–	–	–	–	–
4	42,549,777	1.0068	1.0042	1.0110	1.0029	1.0060	1.0089	–
**4**	**146,211,844**	1.0252	1.0448	1.0712	1.0261	1.0553	1.0829	1.0312
11	17,086,381	1.0076	–	–	1.0014	1.0029	1.0042	–
11	132,309,606	0.9965	–	–	–	–	–	–
12	19,770,625	–	–	–	1.0045	1.0094	1.0140	–
12	47,866,802	1.0184	1.0001	1.0184	–	–	–	1.0140
12	47,870,741	–	–	–	1.0152	1.0320	1.0477	–
14	24,345,235	1.0013	–	–	–	–	–	–
16	81,155,867	–	–	–	1.0039	1.0082	1.0122	–
17	46,592,346	–	–	–	1.0025	1.0052	1.0077	–
17	52,888,414	–	–	–	1.0005	1.0011	1.0017	–
17	69,061,947	–	–	–	1.0021	1.0043	1.0064	–
18	36,210,549	–	–	–	1.0186	1.0392	1.0585	–
19	37,070,882	–	–	–	1.0020	1.0042	1.0062	–
21	32,760,615	–	–	–	1.0051	1.0107	1.0159	–

*Note:* All three methods selected the imputed insertion/deletion (indel) polymorphism on chromosome 4 at position 146,211,844 (rs5862730), which was the only reported SNP that reached genome-wide significance in the full OPPERA cohort.

SNPs: single nucleotide polymorphisms; GEI: gene-environment interaction; TMD: temporomandibular disorder; OPPERA: Orofacial Pain: Prospective Evaluation and Risk Assessment.

Moreover, we present in Table 7, the AUC in the training and test cohorts, the number of predictors selected in each model and the total computation time to fit each method. We see that *pglmm* has the highest AUC on the training data, as well as the best predictive performance on the CPPC cohort alone. On the other hand, *glmnet* and *gesso* both have a greater predictive performance in the OPPERA2 cohort compared to pglmm. When combining the predictions for OPPERA2 and CPPC cohorts, all three methods have similar predictive performance. In term of the number of predictors selected by each model, *glmnet* has selected two SNPs with important main effects and no GEI effects, while *gesso* has selected the highest number of predictors, that is a total of 13 SNPs with both main and GEI effects. On the other hand, our proposed method *pglmm* has selected a total of seven SNPs, among which three had a selected GEI effect with sex. Finally, we report for each method the computational time to fit the model on the training cohort using 10-folds cross-validation. While *glmnet* only took two hours to fit, it failed to retrieve any potentially important GEI effects between TMD and sex, albeit we note that it had a similar predictive performance than the hierarchical methods on the combined test sets. On the other hand, *pglmm* had the highest computational time required to fit the model, because it requires iteratively estimating a random effects vector of size n=3030 , while both *glmnet* and *gesso* only require to estimate a vector of fixed effects of size 10 for the PCs. However, *pglmm* had the highest AUC on the train set, and was able to retrieve potentially important GEI effects for some of the select SNPs in the model, while selecting half as many predictors than gesso.

**Table 7.** Area under the ROC curve (AUC), model size and computational time for the analysis of TMD.

	AUC ${}_{train}$	AUC ${}_{test}$			Model size		
Method	OPPERA	OPPERA2	CPPC	OPPERA2+CPPC	Main effects	GEI effects	Computational time (hours)
*glmnet*	0.722	0.587	0.632	0.551	2	0	2
*gesso*	0.725	0.586	0.630	0.551	13	13	9
*pglmm*	0.867	0.512	0.652	0.550	7	3	47

CPPC: Complex Persistent Pain Conditions; GEI: gene-environment interaction; TMD: temporomandibular disorder; OPPERA: Orofacial Pain: Prospective Evaluation and Risk Assessment.

## 5. Discussion

We have developed a unified approach based on regularized PQL estimation, for selecting important predictors and GEI effects in high-dimensional GWAS data, accounting for population structure, close relatedness, shared environmental exposure and binary nature of the trait. We proposed to combine PQL estimation with a CAP for hierarchical selection of main genetic and GEI effects, and derived a proximal Newton-type algorithm with block coordinate descent to find coordinate-wise updates. We showed that for all simulation scenarios, including and additional random effect to account for the shared environmental exposure reduced the FPR of our proposed method for selection of both GEI and main effects. Using the $F_{1}$ score as a balanced measure of the FDR and TPR, we showed that in the hierarchical simulation scenarios, pglmm outperformed all other methods for retrieving important GEI effects. Moreover, using real data from the OPPERA study to explore the comparative performance of our method in selecting important predictors of TMD, we found that our proposed method was able to retrieve a previously reported significant loci in a combined or sex-segregated GWAS.

A limitation of *pglmm* compared to a logistic lasso or group lasso with PC adjustment is the computational cost of performing multiple matrix calculations that comes from incorporating a GSM to account for population structure and relatedness between individuals. These computations become prohibitive when the sample size increases, and this may hinder the use of random effects in hierachichal selection of both genetic and GEI fixed effects in genetic association studies. Solutions to explore in order to increase computation speed and decrease memory usage would be the use of conjugate gradient methods with a diagonal preconditioner matrix, as proposed by Zhou et al.,^34^ and the use of sparse GSMs to adjust for the sample relatedness.^35^

In this study, we focused solely on the sparse group lasso as a hierarchical regularization penalty. Although previous work has shown that using a CAP penalty with a group $L_{\infty }$ norm (iCAP) might perform better than a sparse group lasso penalty for retrieving important interaction terms,^19^ substantive work is needed to develop an efficient algorithm to fit the iCAP penalty in the presence of random effects. It is also important to highlight that for selection of main effects, the sparse group lasso penalty might perform better than the iCAP penalty. Thus, the choice of which group penalty to use should reflect this trade off between improving the selection of main effects versus selection of important GEI effects. Moreover, it is known that estimated effects by lasso will have large biases because the resulting shrinkage is constant irrespective of the magnitude of the effects. Alternative regularizations like the Smoothly Clipped Absolute Deviation (SCAD)^36^ and Minimax Concave Penalty (MCP)^37^ could be explored, although we note that both SCAD and MCP require tuning an additional parameter which controls the relaxation rate of the penalty. Another alternative includes refitting the sparse group lasso penalty on the active set of predictors only, similarly to the relaxed lasso, which has shown to produce sparser models with equal or lower prediction loss than the regular lasso estimator for high-dimensional data.^38^

Another interesting question to address in the context of high-dimensional GLMMs would be to assess the goodness of fit of the selected sparse model. In the context of high-dimensional GLMs, a recent methodology has been proposed to test for any signal left in the residuals after fitting a sparse model in order to assess whether a sparse non-linear model would be more appropriate.^39^ Although there exist graphical and numerical methods for checking the adequacy of GLMMs,^40^ to our knowledge no such procedure has been extended to high-dimensional mixed models. Finally, it would also be of interest to explore if joint selection of fixed and random effects could result in better selection and/or predictive performance. Future work includes tuning the generalized ridge regularization on the random effects,^41^ or replacing it by a lasso regularization to perform selection of individual random effects.^14,42^

## A Appendix

### A.1 Updates for $\tilde{\delta }$

The gradient and Hessian of f(Θ;δ) are given by the following equations:

$$\begin{array}{rl} \nabla_{\delta }\;f(\Theta ;\delta ) & =-{\hat{\phi }}^{-1}U^{⊺}(y-\mu )+\Lambda^{-1}\delta \\ \nabla_{\delta }^{2}\;f(\Theta ;\delta ) & ={\hat{\phi }}^{-1}U^{⊺}\Delta^{-1}U+\Lambda^{-1} \end{array}$$

This leads to the Newton updates

(7)
$$\begin{array}{rl} {\tilde{\delta }}^{\left(t+1\right)} & ={\tilde{\delta }}^{\left(t\right)}-[\nabla_{\delta }^{2}\;f(\Theta |{\tilde{\delta }}^{\left(t\right)}){]}^{-1}\nabla_{\delta }\;f(\Theta |{\tilde{\delta }}^{\left(t\right)})\\ & ={\tilde{\delta }}^{\left(t\right)}+{\left[{\hat{\phi }}^{-1}U^{⊺}\Delta^{-(t)}U+\Lambda^{-1}\right]}^{-1}\left({\hat{\phi }}^{-1}U^{⊺}(y-\mu^{\left(t\right)})-\Lambda^{-1}{\tilde{\delta }}^{\left(t\right)}\right)\\ & ={\left[U^{⊺}\Delta^{-(t)}U+\hat{\phi }\Lambda^{-1}\right]}^{-1}U^{⊺}\Delta^{-(t)}\left(\Delta^{\left(t\right)}(y-\mu^{\left(t\right)})+U{\tilde{\delta }}^{\left(t\right)}\right) \end{array}$$

which requires repeatedly inverting the n×n matrix $\Sigma^{\left(t\right)}:=U^{⊺}\Delta^{-(t)}U+\hat{\phi }\Lambda^{-1}$ with complexity $O(n^{3})$ where n is the sample size. Defining the working vector $\tilde{Y}=X\Theta^{\left(t\right)}+U{\tilde{\delta }}^{\left(t\right)}+\Delta^{\left(t\right)}(y-\mu^{\left(t\right)})$ , where XΘ=Zθ+Dα+Gβ+(D⊙G)γ , the Newton updates in (7) can be rewritten as follows:

$${\tilde{\delta }}^{\left(t+1\right)}={\left[U^{⊺}\Delta^{-(t)}U+\hat{\phi }\Lambda^{-1}\right]}^{-1}U^{⊺}\Delta^{-(t)}\left(\tilde{Y}-X\Theta^{\left(t\right)}\right)$$

which can be equivalently obtained as the solutions to the following generalized ridge WLS problem:

(8)
$${\tilde{\delta }}^{\left(t+1\right)}=\underset{\delta }{\text{argmin }}{\hat{\phi }}^{-1}{\left(\tilde{Y}-X\Theta^{\left(t\right)}-U\delta \right)}^{⊺}\Delta^{-(t)}\left(\tilde{Y}-X\Theta^{\left(t\right)}-U\delta \right)+\delta^{⊺}\Lambda^{-1}\delta$$

Equation (8) is analogous to the PC ridge regression (PCRR) model,^43^ and demonstrates that PCA and MMs indeed share the same underlying model. At last, to solve (8) without repeatedly inverting the n×n matrix $\Sigma^{\left(t\right)}:=U^{⊺}\Delta^{-(t)}U+\hat{\phi }\Lambda^{-1}$ , we propose using a coordinate descent algorithm,^44^ for which each coordinate’s updates are given, for j=1,…,n , by

(9)
$$\tilde{\delta_{j}}\leftarrow \frac{\sum_{i=1}^{n}w_{i}U_{ij}\left({\tilde{Y}}_{i}-X_{i}\Theta^{\left(t\right)}-\sum_{l\neq j}U_{il}\tilde{\delta_{l}}\right)}{\sum_{i=1}^{n}w_{i}U_{ij}^{2}+\hat{\phi }\Lambda_{j}^{-1}}$$

where $w_{i}=\Delta_{ii}^{-(t)}$ .

### A.2 Updates for Θ

Since the objective function in (5) consists of a smooth convex function f(Θ;δ) and a non-smooth convex regularizer g(Θ) , we propose a proximal Newton algorithm with cyclic coordinate descent to find PQL regularized estimates for Θ , in the spirit of the proposed algorithm by Friedman et al.^26^ for estimation of GLMs with convex penalties. Let again XΘ=Zθ+Dα+Gβ+(D⊙G)γ and $\Theta^{\left(t\right)}$ be the current iterate, the iterative step reduces to

$$\begin{array}{rl} \Theta^{\left(t+1\right)} & =\underset{\Theta }{\text{argmin }}\left\{\frac{1}{2s_{t}}{\left\|\Theta -\left(\Theta^{\left(t\right)}-s_{t}{\left[\nabla_{\Theta }^{2}f(\Theta^{\left(t\right)}|\tilde{\delta })\right]}^{-1}\nabla_{\Theta }f(\Theta^{\left(t\right)}|\tilde{\delta })\right)\right\|}_{2}^{2}+g(\Theta )\right\}\\ & =\underset{\Theta }{\text{argmin }}\left\{\frac{1}{2s_{t}}{\left\|\Theta -{\left[X^{⊺}\Delta^{-(t)}X\right]}^{-1}X^{⊺}\Delta^{-(t)}\left({X\Theta }^{\left(t\right)}+s_{t}\Delta^{\left(t\right)}(y-\mu^{\left(t\right)})\right)\right\|}_{2}^{2}+g(\Theta )\right\} \end{array}$$

where $s_{t}$ is a suitable step size. Defining the working vector $\tilde{Y}=X\Theta^{\left(t\right)}+U{\tilde{\delta }}^{\left(t+1\right)}+s_{t}\Delta^{\left(t\right)}(y-\mu^{\left(t\right)})$ , we can again rewrite the minimization problem as a WLS problem where

(10)
$$\begin{array}{rl} \Theta^{\left(t+1\right)} & =\underset{\Theta }{\text{argmin }}\left\{\frac{1}{2s_{t}}{\left\|\Theta -{\left[X^{⊺}\Delta^{-(t)}X\right]}^{-1}X^{⊺}\Delta^{-(t)}\left(\tilde{Y}-U{\tilde{\delta }}^{\left(t+1\right)}\right)\right\|}_{2}^{2}+g(\Theta )\right\}\\ & =\underset{\Theta }{\text{argmin }}\left\{\frac{1}{2s_{t}}\sum_{i=1}^{n}w_{i}{\left({\tilde{Y}}_{i}-X_{i}\Theta -U_{i}{\tilde{\delta }}^{\left(t+1\right)}\right)}^{2}+(1-\rho )\lambda \sum_{j}\|(\beta_{j},\gamma_{j}){\|}_{2}+\rho \lambda \sum_{j}|\gamma_{j}|\right\} \end{array}$$

where $w_{i}=\Delta_{ii}^{-(t)}$ . We use block coordinate descent and minimize (10) with respect to each component of $\Theta =(\theta^{⊺},\alpha^{⊺},\beta^{⊺},\gamma^{⊺}{)}^{⊺}$ . In practice, we set $s_{t}=1$ and do not perform step-size optimization. We present in Appendix 5 the detailed derivations and our block coordinate descent algorithm to obtain PQL regularized estimates for Θ .

### A.3 Strong rule

In modern genome-wide studies, the number of genetic predictors is often very large, and assuming that most of the predictors effects are equal to 0, it would be desirable to discard them from the coordinate descent steps to speed up the optimization procedure. Tibshirani et al.^45^ derived sequential strong rules that can be used when solving the lasso and lasso-type problems over a grid of tuning parameter values $\lambda_{1}\geq \lambda_{2}\geq \lambda_{m}$ , and more details about the derivation of the sequential strong rule for the sparse group lasso can be found by Liang.^46^ Therefore, having already computed the solution ${\hat{\Theta }}_{k-1}$ at $\lambda_{k-1}$ , the sequential strong rule discards the $j^{th}$ genetic predictor from the optimization problem at $\lambda_{k}$ if

$$\sqrt{{\left(G_{j}^{⊺}(y-\mu ({\hat{\Theta }}_{k-1}))\right)}^{2}+{\left(S_{\rho \lambda_{k-1}}((D⊙G_{j}{)}^{⊺}(y-\mu ({\hat{\Theta }}_{k-1})))\right)}^{2}}\leq (1-\rho )(2\lambda_{k}-\lambda_{k-1})$$

where $S_{\lambda }(\cdot )$ is the soft-thresholding function defined as follows:

$$\begin{array}{r} S_{\lambda }(a)=\left\{ \begin{array}{ll} a-\lambda & \text{if }a>\lambda \\ 0 & \text{if }|a|\leq \lambda \\ a+\lambda & \text{if }a<-\lambda \end{array}\right. \end{array}$$

### A.4 Prediction

Our proposed method to calculate prediction scores in individuals that were not used in training the models is presented in this section. In sparse regularized PQL estimation, we iteratively fit on a training set of size n the working LMM

$$\tilde{Y}=X\hat{\Theta }+\tilde{b}+\epsilon$$

where $\hat{\Theta }=\{{\hat{\Theta }}_{k}\neq 0|1\leq k\leq 2p+m+1\}$ is the set of non-null predictors, and $\epsilon =g^{\prime }(\mu )(y-\mu )∼N(0,W^{-1})$ , with $W=\phi^{-1}\text{diag}\left\{\frac{a_{i}}{\nu (\mu_{i})[g^{\prime }(\mu_{i}{)}^{2}]}\right\}$ the diagonal matrix containing weights for each observation. Let ${\tilde{Y}}_{s}$ be the latent working vector in a testing set of $n_{s}$ individuals with predictor set $X_{s}$ . Similar to Bhatnagar et al.,^47^ we assume that the marginal joint distribution of ${\tilde{Y}}_{s}$ and $\tilde{Y}$ is multivariate Normal :

$$\begin{array}{r} \left[\begin{array}{c} {\tilde{Y}}_{s}\\ \tilde{Y} \end{array}\right]∼N\left(\left[\begin{array}{c} X_{s}\hat{\Theta }\\ X\hat{\Theta } \end{array}\right],\left[\begin{array}{cc} \Sigma_{11} & \Sigma_{12}\\ \Sigma_{21} & \Sigma_{22} \end{array}\right]\right) \end{array}$$

where $\Sigma_{12}=\text{Cov}({\tilde{Y}}_{s},\tilde{Y})={\hat{\tau }}_{g}K_{12}+{\hat{\tau }}_{d}K_{12}^{D}$ is the sum of the $n_{s}\times n$ GSMs between the testing and training individuals, and $\Sigma_{22}=\text{Var}(\tilde{Y})=W^{-1}+{\hat{\tau }}_{g}K_{22}+{\hat{\tau }}_{d}K_{22}^{D}$ . It follows from standard normal theory that

$${\tilde{Y}}_{s}|\tilde{Y},\hat{\phi },\hat{\tau },\hat{\Theta },X,X_{s}∼N\left(X_{s}\hat{\Theta }+\Sigma_{12}\Sigma_{22}^{-1}(\tilde{Y}-X\hat{\Theta }),\Sigma_{11}-\Sigma_{12}\Sigma_{22}^{-1}\Sigma_{21}\right)$$

The predictions are based on the conditional expectation $E[{\tilde{Y}}_{s}|\tilde{Y},\hat{\phi },\hat{\tau },\hat{\Theta },X,X_{s}]$ , that is,

$$\begin{array}{rl} {\hat{\mu }}_{s} & =g^{-1}\left(E[{\tilde{Y}}_{s}|\tilde{Y},\hat{\phi },\hat{\tau },\hat{\Theta },X,X_{s}]\right)\\ & =g^{-1}\left(X_{s}\hat{\Theta }+\Sigma_{12}\Sigma_{22}^{-1}(\tilde{Y}-X\hat{\Theta })\right)\\ & =g^{-1}\left(X_{s}\hat{\Theta }+\Sigma_{12}{\left(W^{-1}+{U\Lambda U}^{⊺}\right)}^{-1}(\tilde{Y}-X\hat{\Theta })\right) \end{array}$$

where g(⋅) is the link function and ${U\Lambda U}^{⊺}$ is the spectral decomposition of the GSM for training subjects, with U the n×n matrix of eigenvectors.

### A.5 Proximal Newton method

Defining the working vector $\tilde{Y}={X\Theta }^{\left(t\right)}+U{\tilde{\delta }}^{\left(t+1\right)}+s_{t}\Delta^{\left(t\right)}(y-\mu^{\left(t\right)})$ with suitable step size $s_{t}$ , we can again rewrite the minimization problem as a WLS problem where

(11)
$$\begin{array}{rl} \Theta^{\left(t+1\right)} & =\underset{\Theta }{\text{argmin }}\left\{\frac{1}{2s_{t}}{\left\|\Theta -{\left[X^{⊺}\Delta^{-(t)}X\right]}^{-1}X^{⊺}\Delta^{-(t)}\left({\tilde{Y}}^{\left(t\right)}-U{\tilde{\delta }}^{\left(t+1\right)}\right)\right\|}_{2}^{2}+g(\Theta )\right\}\\ & =\underset{\Theta }{\text{argmin }}\left\{\frac{1}{2s_{t}}\sum_{i=1}^{n}w_{i}{\left({\tilde{Y}}_{i}-X_{i}\Theta -U_{i}{\tilde{\delta }}^{\left(t+1\right)}\right)}^{2}+(1-\rho )\lambda \sum_{j}\|(\beta_{j},\gamma_{j}){\|}_{2}+\rho \lambda \sum_{j}|\gamma_{j}|\right\} \end{array}$$

with $w_{i}=\Delta_{ii}^{-(t)}$ . We use block coordinate descent and minimize (11) with respect to each component of $\Theta =(\theta^{⊺},\alpha^{⊺},\beta^{⊺},\gamma^{⊺}{)}^{⊺}$ . Suppose we have estimates ${\tilde{\theta }}_{l}$ for l≠j , $\tilde{\beta }$ , $\tilde{\gamma }$ , and $\tilde{\delta }$ , it is straightforward to show that the updates for $\theta_{j}$ and α are given by the following equations:

$$\begin{array}{rl} \tilde{\theta_{j}} & \leftarrow \frac{\sum_{i=1}^{n}w_{i}Z_{ij}\left({\tilde{Y}}_{i}-\sum_{l\neq j}Z_{il}\tilde{\theta_{l}}-D_{i}\tilde{\alpha }-G_{i}\tilde{\beta }-(D_{i}⊙G_{i})\tilde{\gamma }-U_{i}\tilde{\delta }\right)}{\sum_{i=1}^{n}w_{i}Z_{ij}^{2}}\\ \tilde{\alpha } & \leftarrow \frac{\sum_{i=1}^{n}w_{i}D_{i}\left({\tilde{Y}}_{i}-Z_{i}\tilde{\theta }-G_{i}\tilde{\beta }-(D_{i}⊙G_{i})\tilde{\gamma }-U_{i}\tilde{\delta }\right)}{\sum_{i=1}^{n}w_{i}D_{i}^{2}} \end{array}$$

Denote the residual $r_{i;-j}={\tilde{Y}}_{i}-Z_{i}\tilde{\theta }-D_{i}\tilde{\alpha }-\sum_{l\neq j}G_{il}\tilde{\beta_{l}}-\sum_{l\neq j}(D_{i}⊙G_{il}){\tilde{\gamma }}_{l}-U_{i}\tilde{\delta }$ . The subgradient equations for $\beta_{j}$ and $\gamma_{j}$ are equal to

$$0\in \left[\begin{array}{c} -\sum_{i=1}^{n}w_{i}G_{ij}\left(r_{i;-j}-G_{ij}\tilde{\beta_{j}}-(D_{i}⊙G_{ij}){\tilde{\gamma }}_{j}\right)\\ -\sum_{i=1}^{n}w_{i}(D_{i}⊙G_{ij})\left(r_{i;-j}-G_{ij}\tilde{\beta_{j}}-(D_{i}⊙G_{ij}){\tilde{\gamma }}_{j}\right)+\rho \lambda s_{t}\partial \|{\tilde{\gamma }}_{j}{\|}_{1} \end{array}\right]+(1-\rho )\lambda s_{t}\partial \|{\tilde{\beta }}_{j},{\tilde{\gamma }}_{j}{\|}_{2}$$

where we define the subgradients

$$u\in \partial \|{\tilde{\gamma }}_{j}{\|}_{1}=\left\{ \begin{array}{ll} \left[-1,1\right] & \text{if }{\tilde{\gamma }}_{j}=0\\ \text{sign}({\tilde{\gamma }}_{j}) & \text{if }{\tilde{\gamma }}_{j}\neq 0 \end{array}\right.;\;v\in \partial \|{\tilde{\beta }}_{j},{\tilde{\gamma }}_{j}{\|}_{2}=\left\{ \begin{array}{ll} \left\{v|\;\|v{\|}_{2}\leq 1\right\} & \text{if }{\tilde{\beta }}_{j}={\tilde{\gamma }}_{j}=0\\ \frac{1}{\|{\tilde{\beta }}_{j},{\tilde{\gamma }}_{j}{\|}_{2}}\left[\begin{array}{c} {\tilde{\beta }}_{j}\\ {\tilde{\gamma }}_{j} \end{array}\right] & \text{otherwise} \end{array}\right.$$

(1) The case ${\tilde{\beta }}_{j}={\tilde{\gamma }}_{j}=0$ implies
  $$\left[\begin{array}{c} \sum_{i=1}^{n}w_{i}G_{ij}r_{i;-j}\\ \sum_{i=1}^{n}w_{i}(D_{i}⊙G_{ij})r_{i;-j}-\rho \lambda s_{t}u \end{array}\right]=(1-\rho )\lambda s_{t}v$$
  Since $\|v{\|}_{2}\leq 1$ , equality of the constraint holds as long as
  $${\left(\sum_{i=1}^{n}w_{i}G_{ij}r_{i;-j}\right)}^{2}+{\left(\sum_{i=1}^{n}w_{i}(D_{i}⊙G_{ij})r_{i;-j}-\rho \lambda s_{t}u\right)}^{2}\leq ((1-\rho )\lambda s_{t}{)}^{2}$$
  Since u∈[−1,1] , a necessary and sufficient condition for ${\tilde{\beta }}_{j}={\tilde{\gamma }}_{j}=0$ being a solution is
  (12)
  $${\left(\sum_{i=1}^{n}w_{i}G_{ij}r_{i;-j}\right)}^{2}+{\left(S_{\rho \lambda s_{t}}\left(\sum_{i=1}^{n}w_{i}(D_{i}⊙G_{ij})r_{i;-j}\right)\right)}^{2}\leq ((1-\rho )\lambda s_{t}{)}^{2}$$
  where $S_{\lambda }(\cdot )$ is the soft-thresholding function defined as follows:
  $$S_{\lambda }(a)=\left\{ \begin{array}{ll} a-\lambda & \text{if}\;a>\lambda \\ 0 & \text{if}\;|a|\leq \lambda \\ a+\lambda & \text{if}\;a<-\lambda \end{array}\right.$$
(2) The case $({\tilde{\beta }}_{j},{\tilde{\gamma }}_{j}{)}^{⊺}\neq 0$ implies
  (13)
  $$\left[\begin{array}{c} \sum_{i=1}^{n}w_{i}G_{ij}(r_{i;-j}-D_{i}G_{ij}{\tilde{\gamma }}_{j})\\ \sum_{i=1}^{n}w_{i}(D_{i}⊙G_{ij})(r_{i;-j}-G_{ij}{\tilde{\beta }}_{j})-\rho \lambda s_{t}u \end{array}\right]=\left(\left[\begin{array}{cc} \sum_{i=1}^{n}w_{i}G_{ij}^{2} & 0\\ 0 & \sum_{i=1}^{n}w_{i}(D_{i}⊙G_{ij}{)}^{2} \end{array}\right]+\frac{(1-\rho )\lambda s_{t}}{\sqrt{{\tilde{\beta }}_{j}^{2}+{\tilde{\gamma }}_{j}^{2}}}I_{2}\right)\left[\begin{array}{c} {\tilde{\beta }}_{j}\\ {\tilde{\gamma }}_{j} \end{array}\right]$$

We have that ${\tilde{\gamma }}_{j}=0$ if $|\sum_{i=1}^{n}w_{i}(D_{i}⊙G_{ij})(r_{i;-j}-G_{ij}{\tilde{\beta }}_{j})|\leq \rho \lambda s_{t}$ since u∈[−1,1] . This implies that

$$\sum_{i=1}^{n}w_{i}G_{ij}r_{i;-j}=\left(\sum_{i=1}^{n}w_{i}G_{ij}^{2}+(1-\rho )\frac{\lambda s_{t}}{|{\tilde{\beta }}_{j}|}\right){\tilde{\beta }}_{j}$$

with the solution being equal to

$${\tilde{\beta }}_{j}=\frac{S_{(1-\rho )\lambda s_{t}}\left(\sum_{i=1}^{n}w_{i}G_{ij}r_{i;-j}\right)}{\sum_{i=1}^{n}w_{i}G_{ij}^{2}}$$

There is no closed-form solution for (13) if both ${\tilde{\gamma }}_{j}$ and ${\tilde{\beta }}_{j}$ are non-null. In this case, we can replace (11) by a surrogate objective function using a MM algorithm.^48^ From the concavity of the $\ell_{2}$ norm $\|\beta_{j},\gamma_{j}{\|}_{2}=\sqrt{\beta_{j}^{2}+\gamma_{j}^{2}}$ , we have the following inequality:

$$\|\beta_{j},\gamma_{j}{\|}_{2}\leq \|\beta_{j}^{\left(t\right)},\gamma_{j}^{\left(t\right)}{\|}_{2}+\frac{1}{2\|\beta_{j}^{\left(t\right)},\gamma_{j}^{\left(t\right)}{\|}_{2}}\left(\|\beta_{j},\gamma_{j}{\|}_{2}^{2}-\|\beta_{j}^{\left(t\right)},\gamma_{j}^{\left(t\right)}{\|}_{2}^{2}\right)$$

from where we derive the MM iterative step

$$\Theta^{\left(t+1\right)}=\underset{\Theta }{\text{argmin }}\left\{\frac{1}{2s_{t}}\sum_{i=1}^{n}w_{i}{\left({\tilde{Y}}_{i}^{\left(t\right)}-X_{i}\Theta -U_{i}\delta^{\left(t+1\right)}\right)}^{2}+(1-\rho )\lambda \sum_{j}\frac{\|\beta_{j},\gamma_{j}{\|}_{2}^{2}}{2\|\beta_{j}^{\left(t\right)},\gamma_{j}^{\left(t\right)}{\|}_{2}}+\rho \lambda \sum_{j}|\gamma_{j}|\right\}$$

Using cyclic coordinate descent, the updates for $\beta_{j}$ and $\gamma_{j}$ are given by the following equations:

$$\begin{array}{rl} \tilde{\beta_{j}} & \leftarrow \frac{\sum_{i=1}^{n}w_{i}G_{ij}\left(r_{i;-j}-D_{i}G_{ij}{\tilde{\gamma }}_{j}\right)}{\sum_{i=1}^{n}w_{i}G_{ij}^{2}+(1-\rho )\lambda \tilde{s_{t}}}\\ \tilde{\gamma } & \leftarrow \frac{S_{\rho \lambda s_{t}}\left(\sum_{i=1}^{n}w_{i}D_{i}G_{ij}(r_{i;-j}-G_{ij}{\tilde{\beta }}_{j})\right)}{\sum_{i=1}^{n}w_{i}(D_{i}G_{ij}{)}^{2}+(1-\rho )\lambda \tilde{s_{t}}} \end{array}$$

where we defined ${\tilde{s}}_{t}=s_{t}/\|\beta_{j}^{\left(t\right)},\gamma_{j}^{\left(t\right)}{\|}_{2}$ . Algorithm 1 summarizes our block coordinate descent (BCD) procedure to obtain regularized estimates for the fixed effects vector $\Theta =(\theta^{⊺},\alpha^{⊺},\beta^{⊺},\gamma^{⊺}{)}^{⊺}$ .
